# Genes That Bias Mendelian Segregation

**DOI:** 10.1371/journal.pgen.1004387

**Published:** 2014-05-15

**Authors:** Pierre Grognet, Hervé Lalucque, Fabienne Malagnac, Philippe Silar

**Affiliations:** 1 Univ Paris Diderot, Sorbonne Paris Cité, Institut des Energies de Demain, Paris, France; 2 Univ Paris Sud, Institut de Génétique et Microbiologie, Bât. 400, Orsay, France; Duke University Medical Center, United States of America

## Abstract

Mendel laws of inheritance can be cheated by Meiotic Drive Elements (MDs), complex nuclear genetic loci found in various eukaryotic genomes and distorting segregation in their favor. Here, we identify and characterize in the model fungus *Podospora anserina Spok1* and *Spok2*, two MDs known as Spore Killers. We show that they are related genes with both spore-killing distorter and spore-protecting responder activities carried out by the same allele. These alleles act as autonomous elements, exert their effects independently of their location in the genome and can act as MDs in other fungi. Additionally, *Spok1* acts as a resistance factor to *Spok2* killing. Genetical data and cytological analysis of Spok1 and Spok2 localization during the killing process suggest a complex mode of action for Spok proteins. *Spok1* and *Spok2* belong to a multigene family prevalent in the genomes of many ascomycetes. As they have no obvious cellular role, *Spok1* and *Spok2* Spore Killer genes represent a novel kind of selfish genetic elements prevalent in fungal genome that proliferate through meiotic distortion.

## Author Summary

Chromosome segregation during meiosis ensures that paternal and maternal chromosomes are equally transmitted to the progeny. Meiotic Drive Elements (MDs) are known to distort this 1∶1 ratio in many animal, plant, and fungal species by killing the gametes not carrying them. Most of the known MDs are complex genetic loci with separate genes for the killing activity and the resistance to said killing. Here, we report in a model fungus on two genes endowed with MD properties previously unreported. Both genes produce a single polypeptide and confer both killing and resistance. They exert their effect irrespective of their position in the genome. They can cross species barriers and promote bias in segregation in other species. As related genes are frequently observed in fungal genomes, we propose that they are representative of a novel kind of selfish genes that propagate by distorting the Mendel laws of segregation.

## Introduction

In many organisms, genetic factors, called Meiotic Drive Elements (MDs), have found ways to break Mendel's laws of heredity. MDs skew the expected 1∶1 ratio in their favor and are thus overrepresented in the progeny after meiosis. They have been observed in metazoans, plants and fungi [1]. They may play a critical role in population behavior, leading to sex ratio distortion and thus decreasing population size [2]. Additionally, fitness can also be altered by MD factors if they are genetically linked to alleles that confer deleterious traits. Investigation of “Segregation Distorter” in Drosophila [3], [4], “t-haplotypes” in mice [5], [6], [7] and the *S5* locus in rice [8], [9] has showed that MDs are composed of at least two linked genes, the distorter that acts as a toxin by disrupting the formation of gametes, and the responder that acts as an antitoxin that protects from the deleterious distorter effects. These genes are generally embedded in large genomic regions devoid of recombination and containing numerous loci that affect positively or negatively meiotic distortion [3], [5]. In mouse and Drosophila, the distorters and responders originate from cellular genes that have acquired new functions [10], [11].

In fungi, MDs are known as Spore killers (Sks) [12]. In *Neurospora*, three Sks have been discovered [13]. They appear to follow rules similar to other known MDs, as they carry two genetically dissociable distorter and responder loci, embedded in a large region devoid of recombination [13]. The molecular basis for killing is unknown in *Sk-2* and *Sk-3*, as the involved distorters have not yet been isolated. However, a resistance gene (responder) to *Sk-2* and *Sk-3* has been identified [14]. This gene, *NCU09151*, is of unknown function and restricted to species closely related to *N. crassa* (e.g., *Sordaria macrospora*). In the pseudo-homothallic fungus *P. anserina*, at least eight Sks have been observed [15]. One of them has been associated with deleterious effects during ascospore formation of the Het-s prion [16]. However, several additional Sks remain uncharacterized [15], including the first one discovered in fungi [17]. Here, we identify the distorters and responders of two *P. anserina* Sks. Unlike previously known MDs, both activities for these Sks are carried out by single genes acting autonomously irrespective of their position in the genome or of the fungal species and whose homologues are prevalent in many fungi.

## Results

### Identification of *Spok1*

**Figure 1:**
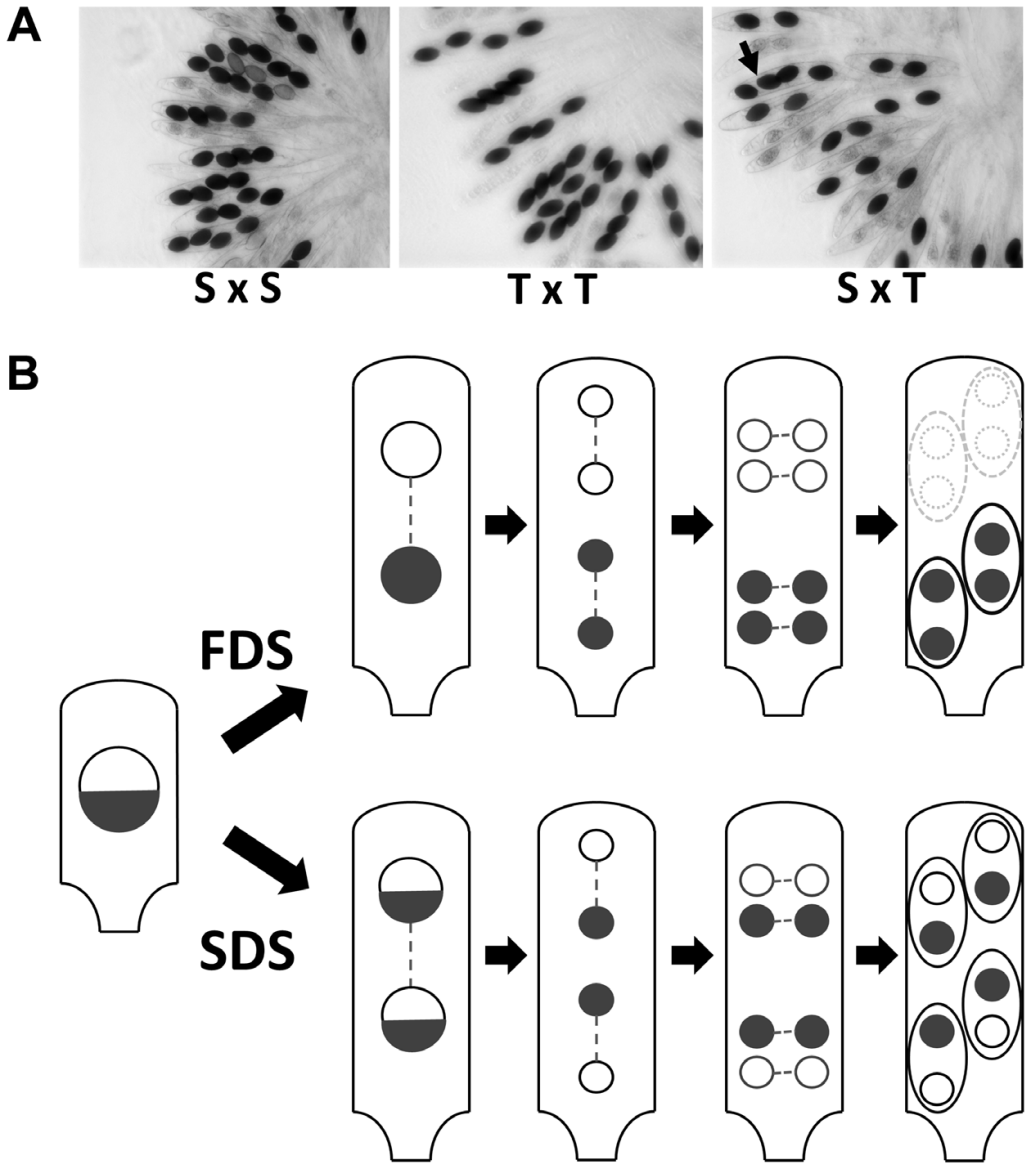
Structure of *P. anserina* asci. (A) S×S or T×T crosses yield 100% four binucleated ascospores per ascus. In S×T cross, 2-spored asci are indicative of spore killing. The Sk locus is linked to the centromere since only 10% of the asci have four ascospores (arrow in S×T). (B) Schematic representation of *P. anserina* FDS and SDS asci with Sk. FDS: no crossover between the Sk locus and the centromere results in First Division Segregation of Sk, triggering death of the two ascospores lacking the Sk locus. SDS: a crossover between the Sk locus and the centromere results in Second Division Segregation of Sk, generating four surviving heterokaryotic ascospores. Proportion of FDS and SDS asci depends upon the frequency of crossover and thus upon the genetic distance between Sk and the centromere. The 90% of 2-spored asci in S×T cross (A) is indicative of a close linkage of the Sk with the centromere.

In crosses between the S and T strains of *P. anserina*, only half the progeny reaches maturity in 90% of the asci (spore sacs; n>200), while all ascospores reach maturity in control S×S and T×T crosses (Fig. 1A). Back cross of the progeny retrieved from S×T crosses to the parental S and T strains showed 90% 2-spored and 100% 4-spored asci, respectively, indicating the presence of at least one Sk in strain T to which strain S is sensitive. It likely corresponds to the first described Sk in fungi [15], [17]. In our hands, this Sk triggers death in nine out of ten asci, and hence harbors a first division segregation (FDS) of 90%, suggesting a close linkage to a centromere (Fig. 1B). During *P. anserina* genome assembly verification by microsatellite genotyping of the progeny from a cross between the S and T strains [18], we observed a strong bias towards the transmission of the T centromere region of chromosome 5 in 50 progeny (Fig. S1) and this was not the case for the other chromosomes, pinpointing the Sk locus close to the centromere of chromosome 5.

**Figure 2:**
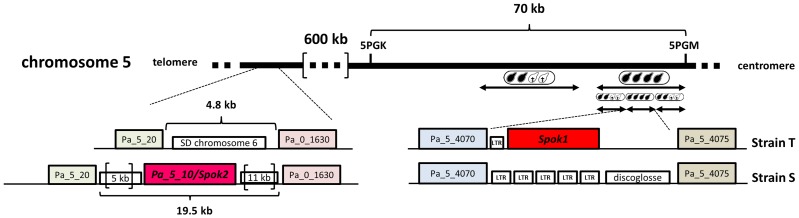
*Spok1* and *Spok2* DNA regions. Double arrows define the sequences deleted to identify *Spok1*. 4-spored asci identify the deletions that abolish spore killing. LTR are Long Terminal Repeats of the crapaud retroposon and discoglosse is a DNA transposon [18]. SD: Segmental Duplication. 5 kb and 11 kb regions bordering *Spok2* contain inactivated transposons. *Pa_x_xxxx* are *P. anserina* predicted CDS.

**Figure 3:**
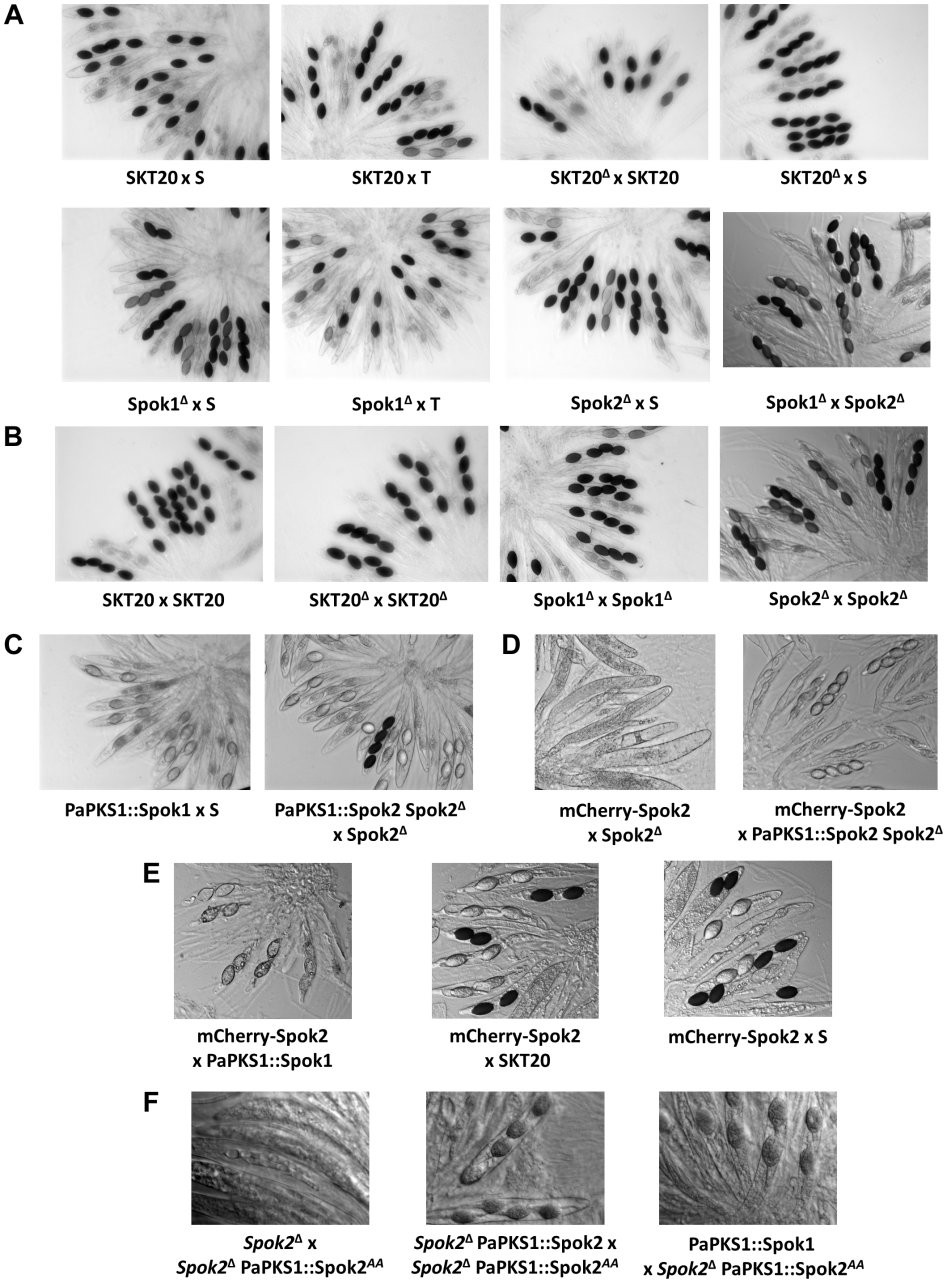
Rosettes of asci in indicated crosses. (A) *Spok1* and *Spok2* are Sks with 90% and 40% FDS, respectively; (B) *Spok1* and *Spok2* have no role in ascospore differentiation; (C) *Spok1* and *Spok2* are functional at the *PaPKS1* locus; the asci with four dark spores results from SDS of the *PaPKS1* locus; (D) complex phenotypes of mCherry-Spok2; (E) *Spok1* and *Spok2* confer resistance to mCherry-Spok2 spore killing; (F) complex phenotypes of Spok2^AA^. See text and table S1 for full genotype of strains.

To narrow the region containing the Sk, we backcrossed a progeny (ST1) of the S×T cross twenty times to strain S, selecting each time for FDS asci. At each generation, we observed the Sk effect (*i.e.*, 90% of 2-spored asci) and thus the final backcrossed strain (SKT20, Table S1) had a genome coming mostly from strain S, except for a small region containing the Sk locus from the T strain. Molecular analysis of polymorphic markers showed that SKT20 had its entire chromosome 5 coming from strain S except for a small region of 70 kb bordered by markers 5PGK and 5PGM (Table S2, Fig. 2). 5PGK differs between S and T for several SNPs that can be detected by sequence analysis. 5PGM differs by the presence of a 15 kb-region present in strain T and absent in strain S. The final backcrossed strain, SKT20, had the 5PGK and 5PGM markers of strain S. This strain displayed 90% 2-spored asci when crossed with S and 100% 4-spored asci when crossed with T, as expected if it contains the distorter and responder of the Sk from strain T (Table 1, Figure 3A). Final identification of the Sk was made by nested deletions in SKT20. A 6 kb-region located between coding sequences (CDS) *Pa_5_4070* and *Pa_5_4075* was found to be responsible for meiotic drive. This region encompassed a retroposon LTR and a single predicted gene, which we called *Spok1* (*Spore killer 1*, Fig. 2, Fig. S2). *Spok1* is absent in strain S, which has transposable elements at the same chromosomal location (i.e., between *Pa_5_4070* and *Pa_5_4075*; Fig. 2, Fig. S2).

**Table 1:** Progeny analysis of Spok crosses.

cross	Spok genotype	progeny	segregation of *Spok2* in progeny
S×S	*Spok2 x Spok2*	100% 4-spored asci	/
T×T	*Spok1 x Spok1*	100% 4-spored asci	/
S×T	*Spok2 x Spok1*	90% 2-spored all with *Spok1*, 10% 4-spored asci	50% *Spok2*: 50% *Spok2^0^*
SKT20×S	*Spok1 Spok2 x Spok2*	90% 2-spored all with *Spok1*, 10% 4-spored asci	/
SKT20×T	*Spok1 Spok2 x Spok1*	100% 4-spored asci	50% *Spok2*: 50% *Spok2^0^*
SKT20×SKT20	*Spok1 Spok2 x Spok1 Spok2*	100% 4-spored asci	/
SKT20^Δ^×SKT20	*Spok1^Δ^ Spok2 x Spok1 Spok2*	90% 2-spored all with *Spok1*, 10% 4-spored asci	/
SKT20^Δ^×SKT20^Δ^	*Spok1^Δ^ Spok2 x Spok1^Δ^ Spok2*	100% 4-spored asci	/
Spok1^Δ^×T	*Spok1^Δ^ x Spok1*	90% 2-spored all with *Spok1*, 10% 4-spored asci	/
Spok1^Δ^×Spok1^Δ^	*Spok1^Δ^ x Spok1^Δ^*	100% 4-spored asci	/
Spok1^Δ^×S	*Spok1^Δ^ x Spok2*	40% 2-spored all with *Spok2*, 60% 4-spored asci	100% *Spok2*
Spok2^Δ^×S	*Spok2^Δ^ x Spok2*	40% 2-spored all with *Spok2*, 60% 4-spored asci	100% *Spok2*
Spok2^Δ^×Spok2^Δ^	*Spok2^Δ^ x Spok2^Δ^*	100% 4-spored asci	/
Spok2^Δ^×T	*Spok2^Δ^ x Spok1*	90% 2-spored all with *Spok1*, 10% 4-spored asci	50% *Spok2^0^*: 50% *Spok2^Δ^*
Spok1^Δ^×Spok2^Δ^	*Spok1^Δ^ x Spok2^Δ^*	100% 4-spored asci	/
SKT20×Spok2^Δ^	*Spok1 Spok2 x Spok2^Δ^*	90% 2-spored all with *Spok1*, 10% 4-spored asci	50% *Spok2*: 50% *Spok2^Δ^*
SKT20 Spok2^Δ^×SKT20	*Spok1 Spok2^Δ^ x Spok1 Spok2*	100% 4-spored asci	50% *Spok2*: 50% *Spok2^Δ^*
SKT20 Spok2^Δ^×SKT20 Spok2^Δ^	*Spok1 Spok2^Δ^ x Spok1 Spok2^Δ^*	100% 4-spored asci	/

*Spok2^0^* corresponds to the locus in strain T devoid of *Spok2* but located at the same chromosomal location.

To validate that *Spok1*, and not an additional non-annotated gene present in the 6-kb region, was necessary and sufficient for spore killing, we first replaced solely its coding sequence with a hygromycin B-resistance marker (Fig. S2, see Materials and Methods for gene deletions and Fig. S3 for Southern Blot validations). The SKT20^Δ^ strain had thus a genotype identical to SKT20, except that the *Spok1* coding sequence was replaced. We observed the production of 100% 4-spored asci in crosses of SKT20^Δ^ with S and 90% 2-spored asci in crosses of SKT20^Δ^ with SKT20 (Fig. 3A), showing that *Spok1* was responsible for both killing and resistance. Secondly, we inserted *Spok1* in the *PaPKS1* gene of strain S. *PaPKS1* is located at the centromere of chromosome 2 and segregates with 99% FDS [19]. It encodes a polyketide synthase that controls the first step of melanin biosynthesis and *PaPKS1* mutants are devoid of pigment at all stages of their life cycle [19]. This allows for easy screening of colorless recombinant transgenic strains carrying an insertion in the *PaPKS1* gene. Additionally, ascospores carrying *Spok1* should be devoid of pigment enabling their easy identification in crosses. Strains carrying *Spok1* at *PaPKS1* (PaPKS1::Spok1) yielded 99% of unpigmented 2-spored asci in crosses with the S strain (Fig. 3C). The 1% 4-spored asci recovered resulted from the expected second division segregation (SDS) of the *PaPKS1::Spok1* locus. Thus, insertion of solely *Spok1* into the *PaPKS1* gene of strain S was sufficient to trigger both spore killing and resistance.

**Figure 4:**
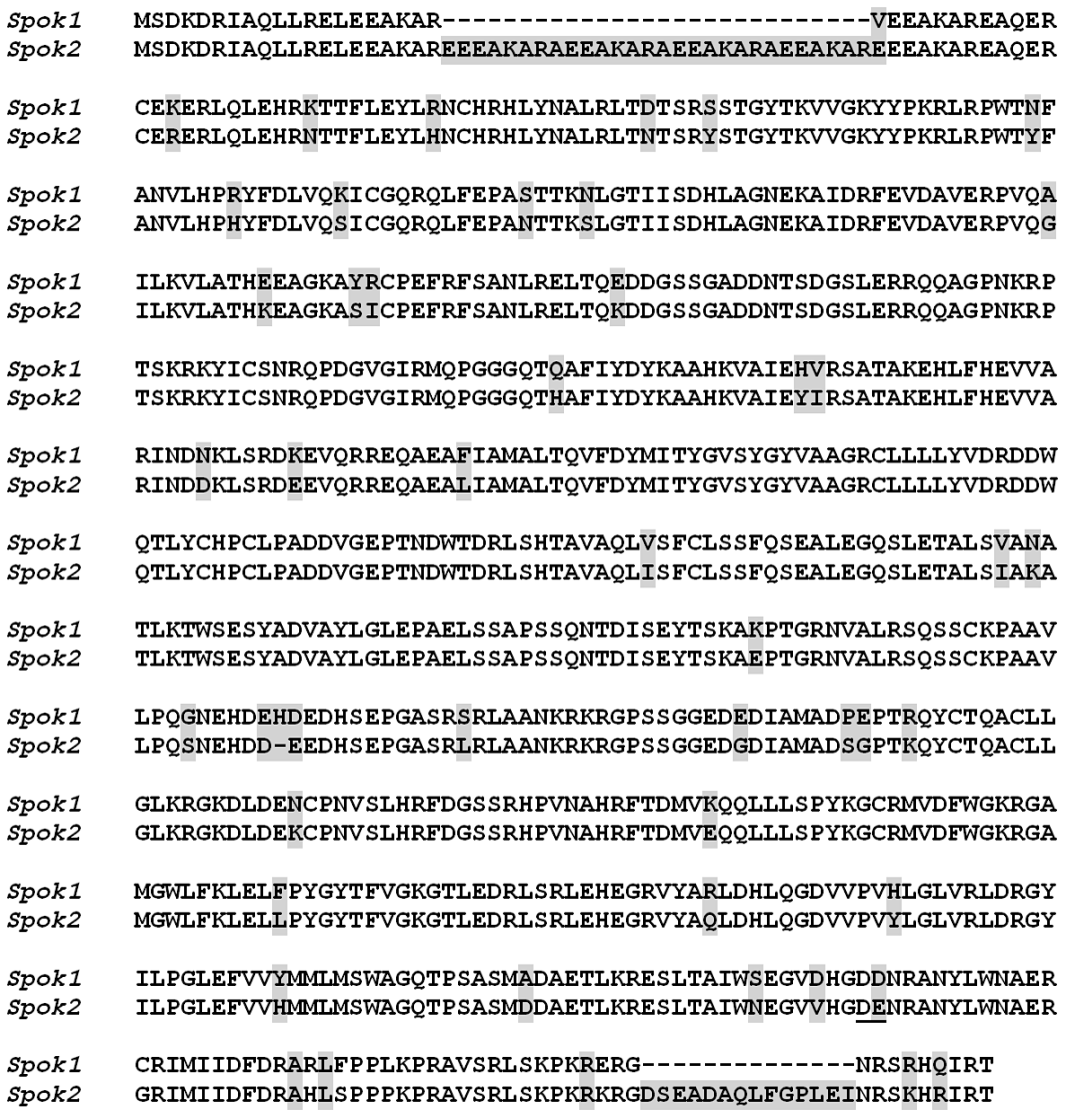
Comparison of Spok1 and Spok2 protein sequences. The amino acids corresponding to the codons changed in the *Spok2^AA^* allele are underlined. Differences between Spok1 and Spok2 are shaded in grey.

Phenotypic analysis of the whole life cycle of SKT20 and SKT20^Δ^ (*i.e.*, ascospore maturation and germination, mycelium growth, heterokaryon incompatibility and sexual reproduction including differentiation of fruiting body) showed no defects other than a lack of Sk activity in SKT20^Δ^, with SKT20×SKT20 and SKT20^Δ^×SKT20^Δ^ homozygous crosses produced 100% 4-spored asci (Table 1, Fig. 3B). Sequence analysis of the 734 amino acid-long Spok1 did not reveal any functional domain (Fig. 4). However, PSORT [20] predicted a nuclear localization.

### Strain S contains *Spok2*, a paralogue of *Spok1*, which also causes spore killing

Surprisingly, when we inactivated *Spok1* in parental strain T (Spok1^Δ^ strain), this did not result in the expected absence of 2-spored asci in Spok1^Δ^×S crosses, as 40% of the asci were 2-spored (Table 1, Fig. 3A). However, the strain yielded 100% 4-spored asci when crossed with itself and 90% 2-spored asci when crossed with strain T, as expected (Table 1, Fig. 3A and 3B). Strain S, but not strain T, carries *Pa_5_10*, a CDS with 87% amino-acid identity to *Spok1* (Fig. 4) and bordered by two large regions composed of Repeat Induced Point mutation (RIP)-inactivated transposons [21] (Fig. 2 and Fig. S2). *Pa_5_10* is 600 kb away from *Spok1* on the same chromosome arm, in a region with an expected FDS of 40% (Fig. 2). In strain T, this position is occupied by a segmental duplication of chromosome 6 (Fig. S2). The *Pa_5_10* gene (hereafter named *Spok2* for *Spore killer 2*) was thus a good candidate for the killing of ascospores in 40% of the asci of the Spok1^Δ^×S crosses. *Spok2* was deleted by replacing its coding sequence with a hygromycin B-resistance marker to yield strain Spok2^Δ^ (Fig. S3). When Spok2^Δ^ was crossed with the parental strain S, 40% 2-spored asci were observed (Fig. 3A). Analysis of the homokaryotic ascospores recovered from a Spok2^Δ^×S cross showed that they were all sensitive to hygromycin B, indicative of a specific killing of the ascospores carrying *Spok2^Δ^* (hygromycin B-resistant) by those carrying wild-type *Spok2*. As expected from its chromosomal location, *Spok2* causes ascospore death in only 40% of asci (n>200; Fig. 3A). Moreover, when the Spok2 coding sequence was inserted in the *PaPKS1* gene of the Spok2^Δ^ strain, using the same strategy as for *Spok1* (PaPKS1::Spok2), it caused ascospore death in 99% of the asci when crossed to the Spok2^Δ^ strain (Fig. 3C). This showed that *Spok2* can also be responsible for spore killing. In crosses between Spok1^Δ^ (strain T) and Spok2^Δ^ (strain S), 100% 4-spored asci were observed (Table 1, Fig. 3A), showing that *Spok2* was responsible for the 40% 2-spored asci present in the Spok1^Δ^×S crosses.

Like *Spok1*, *Spok2* does not appear to be involved in any aspect of the physiology and development of *P. anserina*, as we could not detect any defect in the mycelium, fruiting body and ascospores of the *Spok2^Δ^* strain, with the homozygous cross of this strain yielding 100% 4-spored asci (Table 1, Fig. 3B). Sequence analysis of the Spok2 protein predicted with low probability an ATP binding site of a kinase domain acting on low molecular weight molecules. However, this domain is not predicted for Spok1 despite the great sequence identity (87%) between the two proteins (Fig. 4). Like Spok1, Spok2 was predicted by PSORT to be in nuclei.

### *Spok1* is a resistance factor to *Spok2*

In S×T crosses, we did not detect any obvious meiotic drive created by *Spok2*, i.e., excess transmission of the S genotype in the region surrounding *Spok2* (Fig. S1). This was surprising since in this cross both *Spok1* and *Spok2* are in heterozygous configuration, which should enable killing by both *Spok1* and *Spok2* (Table 1). Possibly, *Spok1* could act as a resistance factor to *Spok2*. To directly test this hypothesis, the 2-spored-asci progeny of *Spok1^Δ^* x S crosses was analyzed. Data showed that all the recovered ascospores had two nuclei containing both the *Spok2* gene (11 asci analyzed), suggesting that *Spok2* exerted spore killing only when the cross was devoid of *Spok1* (Table 1). Homokaryotic “SKT20 Spok2^Δ^” strains, carrying a functional *Spok1* recombined with a deleted *Spok2*, were successfully isolated in the progeny of a cross between SKT20 (which contain functional *Spok1* and *Spok2*; Table 1) and Spok2^Δ^. Upon crossing these SKT20 Spok2^Δ^ strains with SKT20, approximately 40% of homokaryotic descendants (9 of 23) carried the Spok2^Δ^ deletion. Altogether, this showed that *Spok2* triggered spore killing only in the absence of *Spok1*. On the contrary, *Spok2* did not confer any resistance to *Spok1*, as we never obtained homokaryotic progeny that did not carry *Spok1*, in S×T crosses.

### *Spok2* but not *Spok1* is prevalent in *P. anserina* strains

Because *Spok1* and *Spok2* behaved as selfish genetic elements propagating through meiotic drive, we evaluated their presence by PCR amplification of a 630 bp product using primers hybridizing in regions conserved in both *Spok1* and *Spok2* (Table S2) in various strains of *P. anserina* and its sibling species, *P. comata* (Table 2). PCR amplification products were obtained for 19 out of the 22 tested strains. Among the three remaining ones, two (X and CBS411.78) behaved as expected if they lacked both *Spok1* and *Spok2* in crosses with *Spok1*- or *Spok2*- containing strains. The third one (A406) exhibited a surprising behavior, as it was non-killing but resistant to both *Spok1* and *Spok2*. Similar strains with non-killing activities but resistant to all Sks have been found in *N. crassa* [22].

**Table 2:** Spok Sk in *P. anserina* geographic races.

strain	*Spok* 1	S/T^2^	x S	x T	x SKT20	x *Spok1^Δ^*	x *Spok2^Δ^*
A	+	8/25	0	/	90	0	0
B	+	1/18	0	/	90	/	40
D	+	1/18	0	/	90	/	40
E	+	1/18	/	/	/	/	/
F	+	1/18	0	90	90	40	40
H	+	0/19	0	90	90	40	40
I	+	1/18	0	90	90	0	40
M	+	1/18	0	/	90	/	40
N	+	0/19	0	/	90	40	40
s	+	0/19	0	90	90	40	40
R	+	0/19	0	90	90	40	40
U	+	1/18	0	90	90	0	0
V	+	1/18	0	/	90	/	40
W	+	0/19	0	90	90	40	40
X	−	/	50	90	90	0	0
Y	+	14/9	90	100?^3^	90	/	90
Z	+	0/19	0	90	90	40	40
PSCJ14	+	0/19	0	/	90	/	40
PSN14	+	1/18	0	/	90	/	40
A406	−	/	0	0	/	0	0
CBS411.78	−	/	50	/	90	/	0
CBS237.71	+	14/9	90	/	50	/	90

The table gives the percentage of 2-spored asci when crossed with the indicated strain. / means no progeny could be recovered due to sterility of the cross. CBS411.78 and CBS237.11 are labeled as *P. comata* in the Baarn collection, but are fully fertile when crossed with our *P. anserina* strains. PSCJ14 and PSN14 were recently isolated from nature (in 2003 and 2007, respectively). A406 was kindly obtained from D. P. Mahoney and A. E. Bell.

1 presence/absence of a PCR-amplification product when probed with Spok genes-specific primers.

2 number of nucleotide differences with the *Spok* genes of the S and T strains.

3 fertility of this cross is very low and few asci were obtained, all had only two ascospores.

The 630 pb amplification products were sequenced to assess whether they originated from *Spok1* or *Spok2*. *Spok1* was not found in any of the other strains tested here and is thus so far only present in strain T, while analysis of the remaining strains showed that all but two (Y and CBS237.71) contained *Spok2* or a variant of it. Strains B, D, E, F, I, M, U and PSN14 carried a *Spok2* variant with a silent nucleotide polymorphism (A to G at nucleotide N° 1194) and these strains behaved like strain S. Strain A carried another *Spok2* variant with the silent A_1194_ to G substitution, an A to G substitution at position 1029, resulting in a Tyr343 to Cys polymorphism, and a GCCGGT insertion at position 1041 resulting in an insertion of two amino acids (Arg-Cys) after amino acid n°346. This *Spok2* allele was active for resistance to *Spok2* as shown by the recovery of 100% 4-spored asci from A×S crosses. The A×SKT20 crosses yielded 90% of 2-spored asci, showing that *Spok1* was still able to act as a Sk in presence of the *Spok2* allele from strain A. Interestingly, A×Spok1^Δ^ and A×Spok2^Δ^ crosses produced 100% 4-spored asci, showing that this allele was inactive for killing in such crosses (Table 2). Inoperativeness for killing was confirmed by analysis of A×Spok2^Δ^ progeny, in which 50% homokaryotic hygromycin B-resistant ascospores carrying the *Spok2* deletion were present.

*P. comata* CBS237.71 and *P. anserina* strain Y contained the same *Spok*-related gene having 14 and 9 differences with S and T, respectively, in the sequenced region. This gene may be another distinct functional Sk. Indeed, strain Y was previously reported as containing a Sk in crosses with strain S [15]. We confirm this (Table 2), as Y×S crosses presented 90% 2-spored asci. Unfortunately, fertility of strain Y was very low when crossed with strain T and SKT20. However, the few recovered asci suggested a complex interaction between the Sk of strain T and Y, a phenomenon previously seen in crosses with strain O and Us5 [23]. Therefore, it is highly probable that a third *Spok* gene (*Spok3*) endowed with spore killing activity and segregating with 90% SDS is present in Y. *Spok3* could also be present in *P. comata* CBS237.11, which also displays 90% 2-spored asci when crossed with strain S (data not shown). Unfortunately, this strain cannot be crossed with strain T.

### GFP and mCherry tagging alter distorter or responder activities of Spok proteins

To gain some insight into the molecular mechanisms of Spok1 and Spok2 action, we tagged the two proteins at the carboxy- and amino-termini with GFP (Spok1) and mCherry (Spok2). Spok1-GFP and Spok2-mCherry proteins tagged at their C-termini were obtained by introducing the GFP or mCherry CDS upstream of the stop codon of *Spok1* and *Spok2*. GFP-Spok1 and mCherry-Spok2 proteins tagged at their N-termini were obtained by inserting *in vitro* the GFP or mCherry CDS downstream of the *Spok* genes start codons. The chimaeric constructs were then inserted at the *PaPKS1* locus. When crossed with the S, T, SKT20, SKT20*^Δ^*, Spok1*^Δ^* and Spok2*^Δ^* strains, the strains carrying the GFP and mCherry constructs exhibited unexpected patterns. Crosses of Spok1-GFP with all strains yielded 100% asci with four spores, indicating that, while unable to promote killing, the transgene enabled resistance to *Spok1* and *Spok2* killing. Similarly, crosses with Spok2-mCherry showed that this allele was unable to kill *Spok2^Δ^*, yet was resistant to *Spok2*. N-terminally tagged mCherry-Spok2 produced empty asci when crossed with Spok2*^Δ^* (Fig. 3D), as if the transgene conserved the killing activity but lost the resistance one. However, in crosses with the strain having *Spok2* at the *PaPKS1* locus, only 4-spored asci were obtained, as if the responder activity was restored in the presence of a wild-type copy of *Spok2*. Both *Spok1* and *Spok2* enabled resistance to mCherry-Spok2 killing, as crosses with S, SKT20 and PaPKS1::Spok1 yielded ascospores with the expected segregation if full resistance occurred (Fig. 3E). On the contrary, GFP-Spok1 exhibited a pattern of asci expected for a protein endowed with both distorter and responder activities (data not shown). These data indicated that it was possible to independently inactivate either the responder or the distorter activities of Spok proteins, as previously gathered from the variant present in strain A.

### Spok1 distorter and responder domains cannot be easily dissociated

In view of the above results, we tried to determine whether two separate domains carrying either the distorter or the responder activities could be identified in the Spok1 protein. In frame deletions in *Spok1* were made *in vitro* and the truncated genes were reintroduced in the *PaPKS1* gene, as done with full length *Spok1* (Fig. S4). None of the construct carried any functional killing and resistance activity (n>30), showing that the two functions could not easily be separated on two independent DNA fragments.

### Aspartate707/Glutamate708 are important for Spok2 activity

As mentioned above, a putative kinase domain was predicted at the C-terminus of Spok2, but not in Spok1. Pfam analysis [24] identified aspartate707 as a potential catalytic residue in Spok2. Because the next residue (n°708) was a glutamate, which may substitute to aspartate707 in the catalytic center, we mutated both the aspartate707 and glutamate708 to alanines. The recovered mutant, *Spok2^AA^*, was inserted in the *PaPKS1* gene in the Spok2^Δ^ strain. When crossed with Spok2^Δ^, the strain carrying *Spok2^AA^* yielded empty asci, as if the distorter activity was active and the responder one was inactive (Fig. 3F). However, cross of *Spok2^AA^* with the strain carrying a wild-type *Spok2* allele at the *PaPKS1* locus produced asci with four colorless ascospores (Fig. 3F). In such cross, both the *Spok2* and *Spok2^AA^* ascospores survive, as if the *Spok2^AA^* responder activity was active. The behavior of the *Spok2^AA^* allele was thus identical to the one of N-terminally tagged mCherry-Spok2. Finally, when crossed with a strain carrying *Spok1* at the *PaPKS1* locus, asci containing two colorless ascospores were obtained in 99% of the asci, as if *Spok1* acted alone.

### Spok proteins accumulate in nuclei

**Figure 5:**
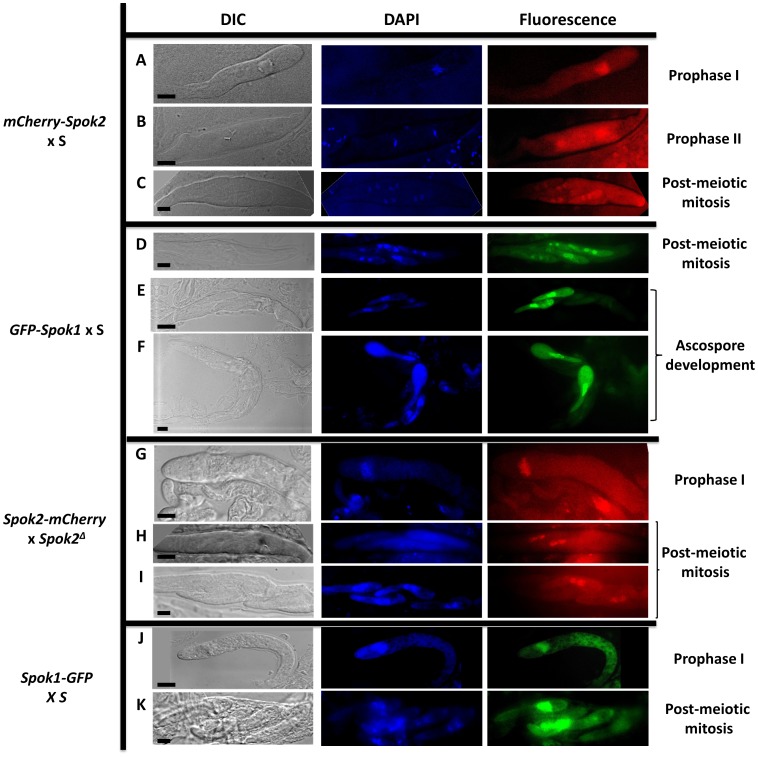
Localization of Spok protein in developing asci. A to F: Localization of N-terminus tagged proteins with maintained spore killing activity. During prophase I, Spok proteins are located mainly in the nucleus and to a lesser extent in the cytoplasm (A & B). After the post-meiotic mitosis, Spok proteins are present in all nuclei (C & D). During ascospore development, sensitive spores are killed and the Spok proteins localize mainly in the nucleus of the resistant spores (E & F). G to K: Localization of the C-terminus tagged proteins without spore killing activity. Fluorescence pattern in prophase I is identical to the one with N-terminus-tagged proteins (G & J). After the post-meiotic mitosis, fluorescence is observed only in the nuclei of the two surviving spores (H, I & K). bar  = 5 µm.

For the four constructs, GFP and mCherry fluorescence was detected in the mycelium and in the fruiting bodies. During ascosporogenesis, similar patterns were observed for all constructs up to the post-meiotic mitosis: a diffuse cytoplasmic presence and an accumulation inside nuclei, as predicted by PSORT (Fig. 5). After this mitosis, at the stage at which two nuclei are present in each ascospore [25] (Fig. 1B), fluorescence was clearly discernible in nuclei of all ascospores in the asci were spore killing occurred (*mCherry-Spok2* x S and *GFP-Spok1* x S crosses). At the beginning of ascospore development, fluorescence was detected in the nuclei of all spores (Fig. 5C and 5D), including those undergoing death. At later stages, fluorescence persisted only in the surviving two ascospores, while the two others degenerated (Fig. 5E and 5F). In asci of crosses where no death occurs (Spok2-mCherry x Spok2*^Δ^* and *Spok1-GFP* x S), we could not detect fluorescence in two out of the four ascospores as early as after post-meiotic mitosis in asci undergoing FDS for *Spok* genes (Fig. 5H, 5I, 5K), suggesting that lack of death in these crosses was due to reduced levels of Spok1 or Spok2 in sensitive ascospores.

### The genomes of *P. anserina* and other fungi contain genes related to *Spok1* and *Spok2*

**Figure 6:**
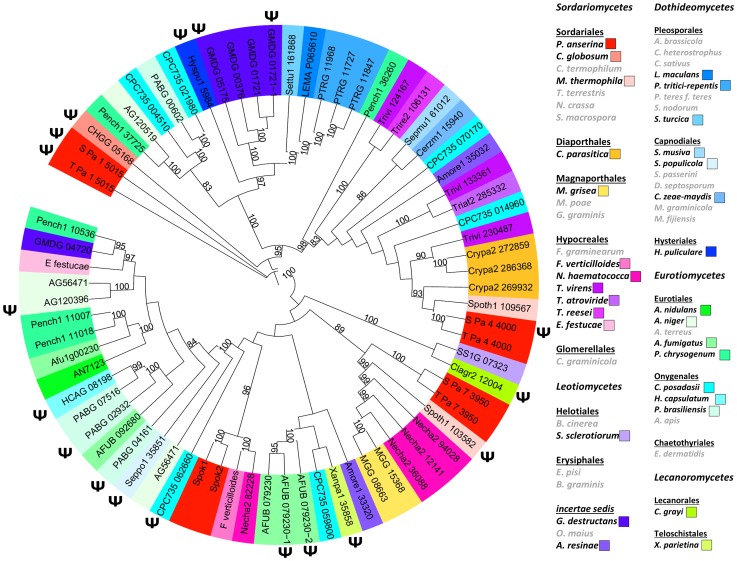
Phylogenetic tree of *Spok* and *Spok*-like genes in representative fungal species. ψ: putative pseudogenes. Right: species in grey: no detected *Spok* or *Spok*-like gene and pseudogene. The other species were color coded according to the known phylogeny.

Mining available databases of complete genome sequences showed that homologues of *Spok* genes are present and prevalent in the genomes of many filamentous ascomycetes (spore sac fungi; Fig. 6). They are present in all major classes of *Pezizomycotina* except in the basal classes *Orbiliomycetes* and *Pezizomycetes*, but in a patchy distribution with closely related species having or lacking *Spok*-related genes. Numbers can go up to 9 and 11 *Spok*-like genes in the genomes of *Fusarium oxysporum* and *Microsporum canis*, respectively. Interestingly, tree construction with selected species showed that the *Spok*-like gene phylogeny did not follow the known evolution of fungi, indicative of possible horizontal transfers (Fig. 6). Moreover, they were often present as pseudogenes, identified by the presence of mutations interrupting the coding sequence or by truncation. *P. anserina* contains three more *Spok*-like genes (*Pa_7_3950*, *Pa_4_4000* and *Pa_1_5015*), all with transposable elements in their vicinity. They are all present in S and T, each occupying the same locus in both strains.

### *Spok* triggers spore killing in other fungi

**Figure 7:**
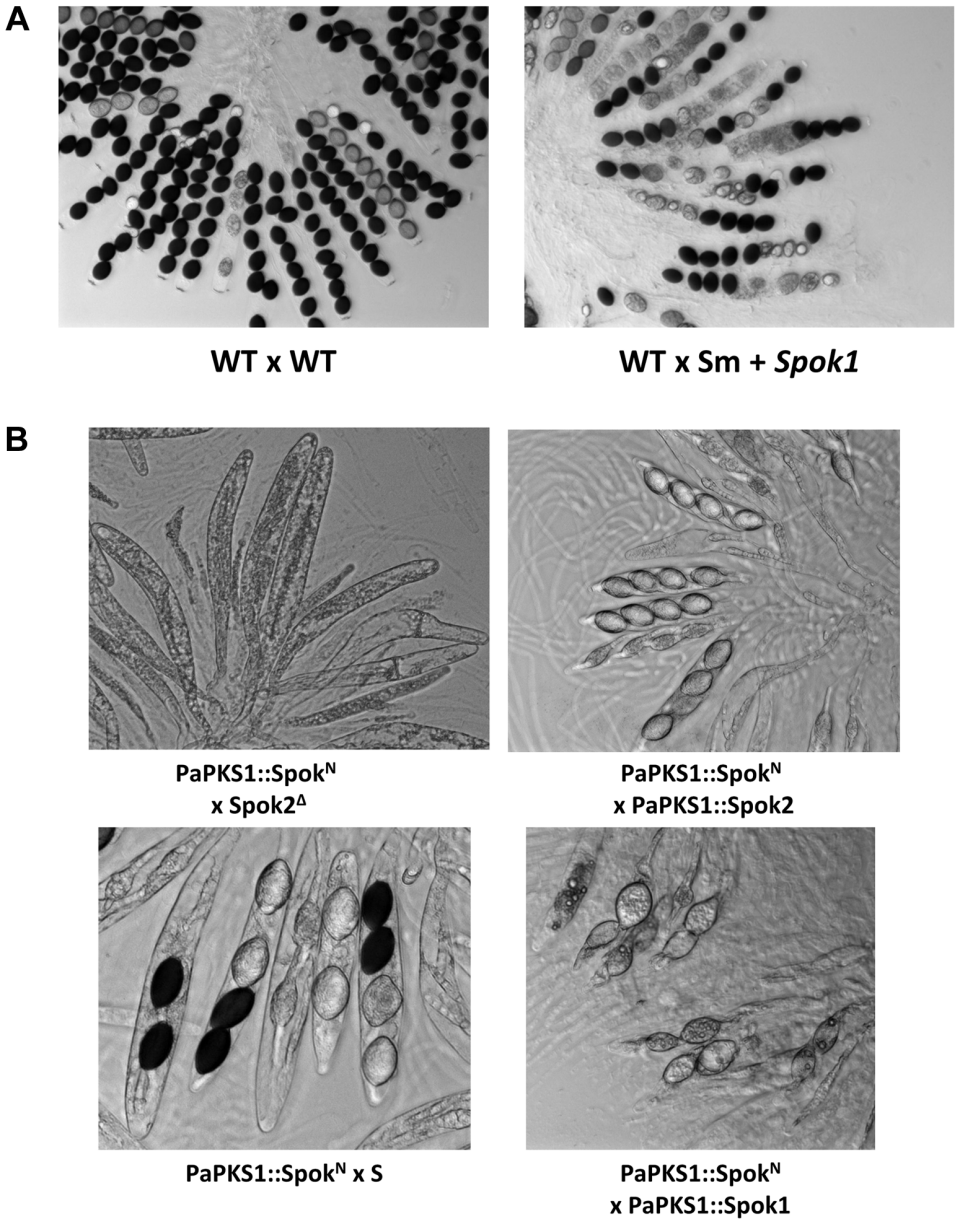
Heterologous expression of *Spok* genes. (A) *S. macrospora* crosses showing Sk activity of *Spok1* in another species. (B) Expression of *Spok^N^* at the *PaPKS1* locus of the Spok2^Δ^ strain results in empty asci, showing that heterologous expression of Spok^N^ results in efficient killing and lack of resistance to said killing. As for mCherry-Spok2 and Spok2^AA^, crosses with PaPKS1::Spok2 result in 4-spored asci; PaPKS1::Spok^N^ x PaPKS1::Spok1 and PaPKS1::Spok^N^ x S crosses showed that *Spok1* and *Spok2* promote resistance to *Spok^N^*, respectively.

To determine whether *Spok1* was able to trigger ascospore death in another species, it was introduced under the expression of its own promoter in *Sordaria macrospora* along with a hygromycin B-resistance marker. This fungus is related to *P. anserina*, even if the genetic distance (i.e., average percentage identity between orthologous proteins) between *Laesiophaeriaceae* to which *P. anserina* belongs and *Sordariaceae* to which *S. macrospora* belongs is equivalent to that between mammals and fishes [18]. It is homothallic and ascospore morphogenesis is different, as eight ascospores are differentiated around single nuclei [26]. Genome sequence analysis indicates that *S. macrospora* is devoid of *Spok* genes (Fig. 6). Eight transformants carrying *Spok1* were recovered and crossed to a strain devoid of *Spok1*. Resulting asci contained four wild-type-looking darkly-pigmented spores and four smaller often-abnormal unpigmented spores (Fig. 7). Wild-type-looking and abnormal ascospores were germinated and tested for resistance to hygromycin B and spore killing activity. 45 out of 66 wild-type-looking ascospores germinated, all were resistant to hygromycin B. 17 were successfully crossed to the strain devoid of *Spok1*. All showed a segregation of four normal and four abnormal ascospores. Two white ascospores out of 66 germinated. Both were resistant to hygromycin B. One was successfully crossed to the strain devoid of *Spok1*. Progeny was composed of asci with four wild-type-looking spores and four abnormal spores. The two germinated spores contained *Spok1* and likely corresponded to unripe ascospores devoid of pigments, as sometime seen in crosses. Therefore, *Spok1* is able to create meiotic drive in *S. macrospora*.

Finally, we introduced the *Necha2_82228* gene from *Nectria haematococca* (Fig. 6) into the *PaPKS1* gene of strain Spok2^Δ^, with its own promoter and terminator sequences. Despite being the closest relative of Spok1/Spok2 on the tree of Fig. 6, the Spok^N^ protein is only 34% and 33% identical to Spok1 and Spok2, respectively. Note that the evolutionary gap between *P. anserina* and *N. haematococca*, is much larger than that between *S. macrospora* and *P. anserina*, arguing for strong differences in modality of ascospore differentiation [27]. PaPKS1::Spok^N^ x Spok2^Δ^ crosses were barren, as all asci were empty, indicating that the Spok^N^ protein had a non-autonomous killing action, reminiscent of the Spok2^AA^ and mCherry-Spok2 proteins that kills but does not allow for resistance. As for these two proteins, PaPKS1::Spok^N^ x PaPKS1::Spok2 crosses produced 4-spored asci. Likewise, PaPKS1::Spok^N^ x PaPKS1::Spok1 and PaPKS1::Spok^N^ x S crosses showed that *Spok1* and *Spok2* promote resistance to *Spok^N^*, respectively. Therefore, despite the great divergence between Spok^N^ and Spok1/Spok2, killing activity is retained and meiotic drive could be promoted by linking genetically *Spok^N^* with a resistance factor such as *Spok1* or *Spok2*.

## Discussion

The *Spok* MDs that we report here are constituted of single genes that carry both distorter and responder activities, unlike those of animals, plants and *Neurospora*, which are large and complex loci with two major genes, the distorter and the responder, and several additional ones that quantitatively modify the effects of either the distorter or the responder [3], [5], [6], [8], [14]. Unlike other MDs, *Spok* MDs may not have originated from cellular genes that have acquired additional functions disrupting normal gamete/spore formation, as they do not appear to be endowed with any physiological function. In *P. anserina*, another MD has been linked to the Het-s prion [16], in which spore killing results from an incompatibility reaction triggered by the co-presence of both the het-s protein in a prion-aggregated form and the soluble Het-S protein. A similar reaction is observed in vegetative hyphae when the two proteins are mixed together. From a genetic point of view, both the *Het-s* and *Het-S* alleles need to be present in crosses to see the MD effects. *Spok* genes act differently from the *Het-s*/*Het-S* MD, since their mere presence at a locus is sufficient to trigger the preferential transmission of this locus during meiosis. *Spok* genes thus define a new class of selfish elements that propagate vertically *via* meiotic drive and possibly horizontally in association with mobile elements. Indeed, at least in *P. anserina*, *Spok*-like genes are always in the vicinity of transposons and they do not seem to play any role in normal development.

Bioinformatic analysis did not provide many clues regarding the potential mode of action of *Spok* genes. An ATP-binding site of a kinase domain was predicted at the C-terminus of Spok2 with low probability, but not in Spok1, questioning its validity. A first model based on the presence of such a domain to explain the dual activity of the Spok genes can be put forward as follows. Spok proteins could be bifunctional enzymes that catalyze both the formation of a toxin from cellular metabolites and its inactivation. The toxin could diffuse in the ascus, while the enzyme and hence the detoxifying activity could not. This should result in the death of the ascospores not expressing the Spok proteins. If the kinase domain is involved in the formation of the toxin, its mutation should result in an allele inactive for killing but active for resistance. On the contrary, if the kinase domain is involved in detoxification, the mutation should produce an allele active for killing and inactive for resistance. However, our data show that this model is unlikely since the mutation of the putative catalytic residues, Aspartate707/Glutatmante708, has a more complex effect as it creates a *Spok2^AA^* allele unable to resist the toxin it produces, but which is fully resistant in the presence of a wild-type *Spok2* allele in the cross. Therefore, it is as if wild-type Spok2 could activate the responder activity in Spok2^AA^, while Spok2^AA^ could not. A second model for the dual mode of action of Spok proteins could be inspired from the yeast killer toxins for which the preprotoxin is a precursor of the toxin but confers resistance by complex formation with the toxin and subsequent degradation [28]. In yeasts, the preprotoxin genes are carried by double-stranded RNA viruses and are not known to trigger meiotic drive. Although, *Spok1* and *Spok2* do not present any obvious sequence similarity with the yeast killer toxin genes, we could propose that they would operate in a similar manner in which the preprotoxin (responder) is involved in resistance to the toxin (distorter). In such model, killing and resistance will depend on a subtle balance between toxin production from preprotoxin processing and preprotoxin/toxin complexes degradation. This may account for all the features presented by *Spok* Sks, including cross-resistance triggered by *Spok1* to the *Spok2* Sk (Spok1 could inactivate the Spok2 toxin, while Spok2 could not remove the Spok1 toxin), the inability to separate two domains by deletion analysis as well as the phenotype of the mCherry-Spok2, Spok2^AA^, Spok^N^ alleles. Indeed, interactions between the Spok2 preprotoxin or toxin and the mCherry-Spok2, Spok2^AA^, Spok^N^ proteins could result in their rapid degradation leading to inability to produce sufficient amounts of toxins. Cytological observations are also compatible with this model as GFP- and mCherry-tagged proteins that do not promote killing disappear very early during ascus maturation. A last model would posit that the Spok proteins may be the toxins and the *Spok* genes the responders that would inactivate the toxins by sequestering them at a defined place inside the nucleus. This would require protein/DNA as well as protein/protein interaction to allow the binding of many toxin molecules on few DNA sequences. In this model, the putative ATP binding site could thus be involved in DNA (nucleotide) binding rather than in a kinase activity. In this model, the Spok2 protein could bind the *Spok1* gene, while the converse would not be possible explaining the resistance of Spok1 over Spok2 effects. The mCherry-Spok2, Spok2^AA^, Spok^N^ proteins would be unable either to enter the nucleus or bind directly the *Spok2*/*Spok^N^* gene, but could do so in the presence of the wild-type Spok2 protein through protein/protein interactions. It is also compatible with the nuclear localization of the Spok proteins after delimitation of the ascospores.

*Spok1* and *Spok2* have many similar homologues in a wide array of filamentous ascomycetes, including in *P. anserina* itself. These are present in a patchy phylogenetic distribution, even in the *P. anserina* populations, do not follow the known fungal evolution and are often present as pseudogenes. The hypothesis that meiotic drive elements are invasive, can result in fewer progeny and can transport bad hitchhikers may explain both the unusual phylogeny and pseudogenes. The fact that a *Spok*-like gene can be a resistance factor to other *Spok*-like Sks also complicates the evolution of this family of genes. As *Spok1* alone is able to trigger meiotic drive in *S. macrospora* and because *Spok^N^* may do so in *P. anserina* when associated with a *Spok* resistance factor(s), we surmise that *Spok*-like genes may account, in part, for the additional Sks detected in many other fungi, including *P. anserina* itself.

## Materials and Methods

All protocols for cultivation, genetic and molecular analysis with *P. anserina* are available at http://podospora.igmors.u-psud.fr. Similar culture techniques were used for *S. macrospora*. Crosses were performed on M2 minimal medium using the S-derived strains as females and T-derived strains as males (supplementary Table S1). Sk was detected by the presence of 2-spored asci in the F1 progeny and assignments to *Spok1* or *Spok2* were made by measuring the ratio of 2-spored versus 4-spored asci and by backcrossing the F1 progeny to the S, T, *Spok1^Δ^* and *Spok2^Δ^* strains and observing the F2 progeny. The sequence of *Spok1* has been deposited in GenBank with accession n° JX560967.

### Polymorphic marker analysis

Genomic DNA was extracted from 50 progenies of an S×T cross (ST1 to ST50). Markers were amplified by PCR with 5 min denaturation at 94°C followed by 30 cycles [30 sec 94°C, 30 sec 55°C, 1 min 72°C] and finished by 10 min elongation at 72°C. The primer pairs used are given in Supplementary Table S2. DNA was separated on 2% agarose gels. DNA extracted from the parental S and T strains was used as a control.

### Deletion

*Spok1* and *Spok2* deletions were made on strains *TΔmus51* and *SΔmus51*, respectively, as described for PaTLK2 in [29] using Hygromycin B resistance as a selection marker. Table S2 gives the primers used for deletions. The deletions were verified by Southern blotting as in [30].

### Insertion of *Spok* alleles at the centromere of chromosome 2

Insertion of *Spok* alleles was made with a strategy involving integration of a plasmid with a single crossover into the *PaPKS1* gene resulting in its inactivation. A 1395 bp DNA fragment from *PaPKS1* was amplified by PCR using S genomic DNA and primers 193SkFSII and 193SkRSI. A 4344 bp DNA fragment surrounding *Spok1* was amplified by PCR with the *Pfu* DNA polymerase from Promega (Madison, WI, USA) using T genomic DNA and primers 510FSI and 510RNI. The *PaPKS1* fragment was digested with *Sal*I and *Sac*II enzymes, the *Spok1* fragment with *Sal*I and *Not*I and both were ligated into pBC-phleo vector [31] cut with *Sac*II and *Not*I to yield pEnterprise1. pEnterprise1 was introduced by transformation into the *SΔmus51::su8-1* strain and one transformant devoid of pigment, was selected for further analysis. *Spok2* and *Spok^N^*, including their own promoters and terminators, were fused by PCR with the 1395 bp *PaPKS1* DNA fragment. The fused PCR fragments was digested with *Sac*II and *Not*I and cloned into pBC-phleo and pBC-Genet vectors to yield pEnterprise2 and pEnterpriseNectria, respectively. Both plasmids were then introduced into *P. anserina* as for pEnterprise1. The same strategy was used to introduce *GFP-Spok1* and *mCherry-Spok2* and the truncated alleles (used primers in Table S2).

### Creation of the *Spok2^AA^* allele

To create the *Spok2^AA^* allele, the plasmid pEnterprise2 was amplified by PCR using primers Spok2MutF and Spok2MutR (Table S2, in bold red are the nucleotides used to change the aspartate and glutamate codons to alanine ones). The PCR product was transformed into *Escherichia coli* and candidates were selected by sequencing the mutated region. One candidate was completely sequenced, found devoid of mutations and introduced in a *Spok2^Δ^ Δmus51* strain. Two transformants were selected based on their lack of pigments and used for further analysis.

### Insertion of *Spok1* in *S. macrospora*

The *Spok1* fragment was excised from pEnterprise1 and cloned into the pBC-Hygro plasmid cut with *SalI* and *NotI*. The pBC-Hygro containing *Spok1* plasmid was transformed into the *spo11* mutant of *S. macrospora*. The wild-type strain and 27 hygromycin B-resistant transformants were crossed with the *spo55* mutant to force outcrossing.

### Microscopy analysis

Perithecium contents for fluorescence analysis were prepared as in [26]. Pictures were taken with a Leica DMIRE 2 microscope coupled with a 10-MHz Cool SNAPHQ charge-coupled device camera (Roper Instruments). They were analyzed with ImageJ.

### Phylogenetic analysis

The phylogenetic analysis of Supplementary Fig. S2 was carried out by aligning the sequences with MAFFT [32] and trimming them with Jalview to retain informative positions [33]. The tree was constructed using PhyML [34] with the default parameters [35] and 100 bootstrapped data sets. The tree was visualized with the iTOL server [36]. Pseudogenes were defined by the presence of several mutations (deletions, frameshifts or read-through) inactivating the coding sequences.

## Supporting Information

Figure S1 Preferential transmission of T markers near the centromere (red dot) of chromosome 5 in 50 descendants of S x T cross. Letters indicate the parental origin of the markers in the progeny strains. Positions of markers on chromosome 5 are indicated at the top. Bracket defines the region with strongly biased transmission of T markers around 5PH1. The red arrow marks the position of *Pa_5_10* (*Spok2*) in the genome of strain S.
(TIF)

Figure S2 Comparison of *Spok1* and *Spok2* loci in strains S and T. The comparisons were drawn with the ACT genome comparison tool [37]. Identical regions are linked by red connections. *Spok* genes are in green. Neighboring genes are in light blue and mobile elements in other colors. The red lines depict the different deleted regions.
(TIF)

Figure S3 Southern blot analysis of SKT20^Δ^, Spok1^Δ^ and Spok2^Δ^ strains. Genomic DNA was extracted from the indicated strains and cut with appropriate restriction enzymes. (A) and (C) predicted structures of *Spok1* and *Spok2* loci before and after marker replacement. (B) and (D) results of Southern blots showing the expected bands. The DNA fragment labeled with * were used as probe.
(TIF)

Figure S4 Deletions analysis of *Spok1*. Schematic representation of the six truncated alleles introduced at the *PaPKS1* locus. Codon numbers are indicated. In all constructs, the *Spok1* promoter, terminator, start and stop codons were retained.
(TIF)

Table S1 Strains used in this study.
(DOCX)

Table S2 Primers used for polymorphic marker analysis, gene deletions and cloning.
(DOCX)
