# Supplementary figures and images for: Genes That Bias Mendelian Segregation
Source: PLoS Genet. 2014 May 15;10(5):e1004387. doi: 10.1371/journal.pgen.1004387 (PMC4022471; doi:10.1371/journal.pgen.1004387)

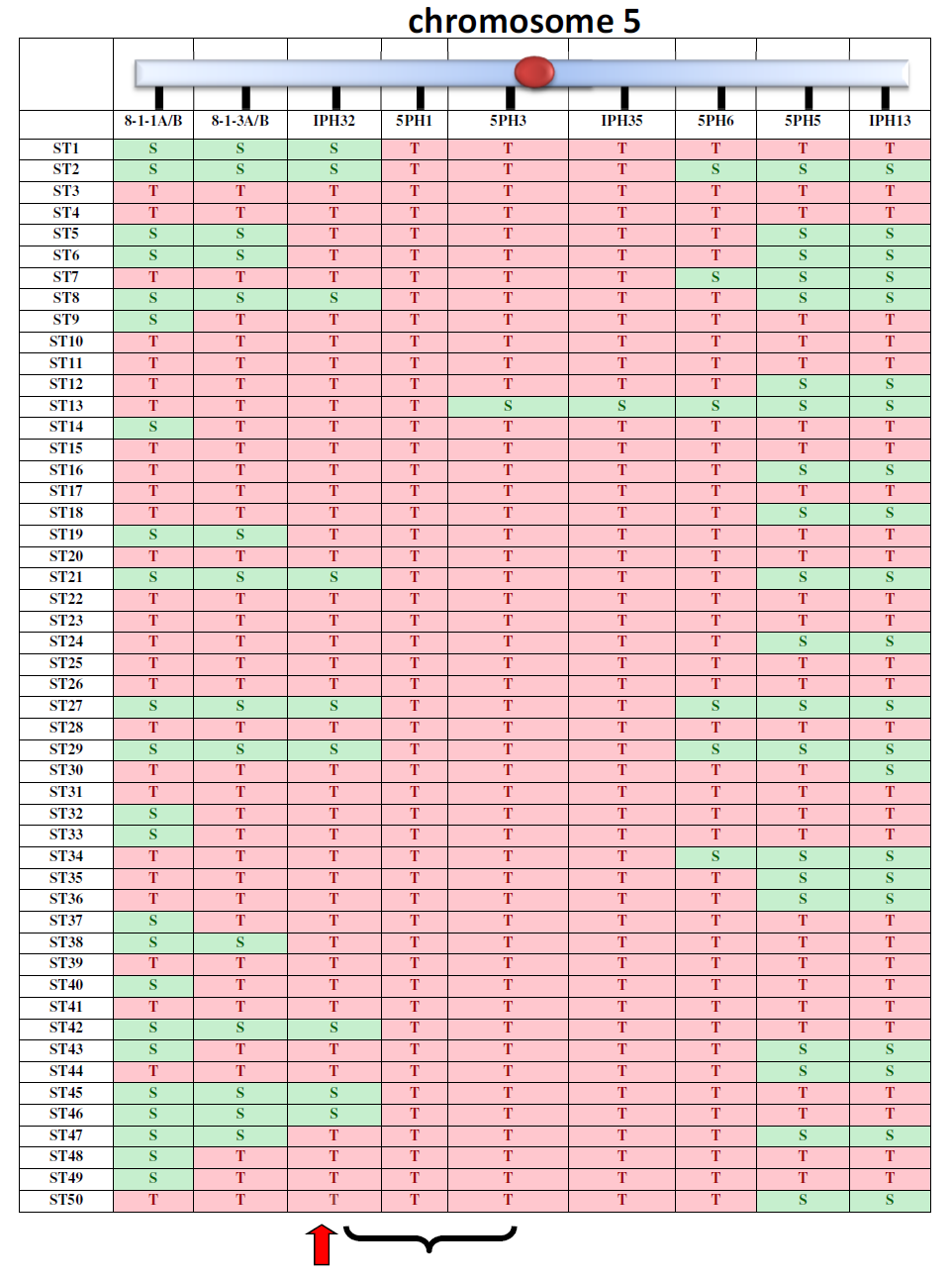

Supplement: Figure S1 — Preferential transmission of T markers near the centromere (red dot) of chromosome 5 in 50 descendants of S x T cross. Letters indicate the parental origin of the markers in the progeny strains. Positions of markers on chromosome 5 are indicated at the top. Bracket defines the region with strongly biased transmission of T markers around 5PH1. The red arrow marks the position of Pa_5_10 (Spok2) in the genome of strain S. (TIF) [file pgen.1004387.s001.tif]

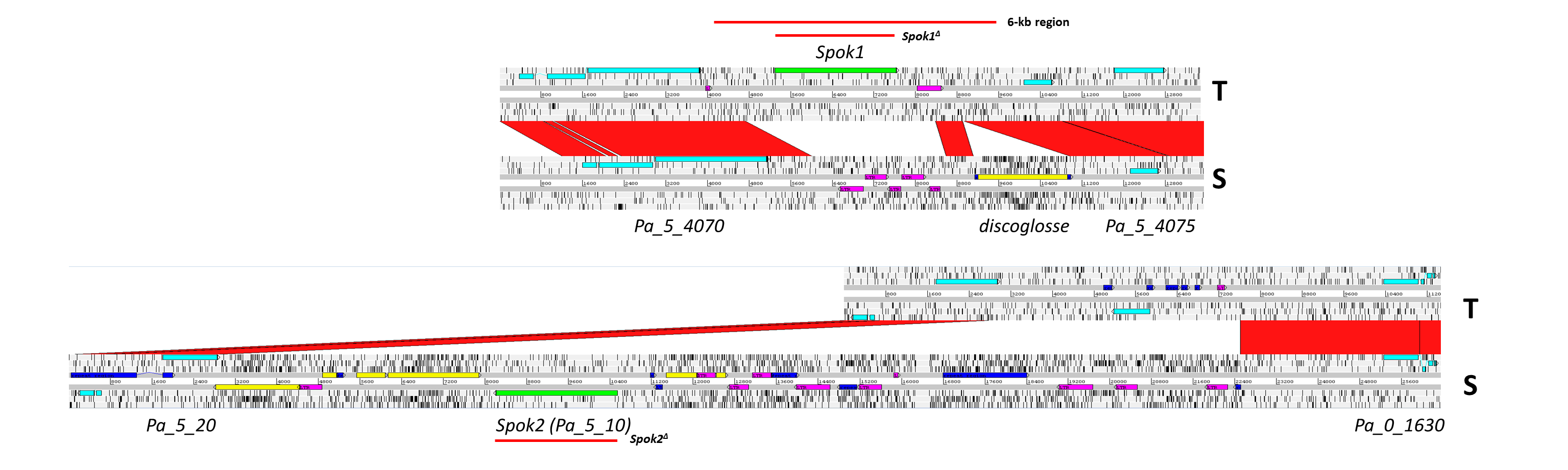

Supplement: Figure S2 — Comparison of Spok1 and Spok2 loci in strains S and T. The comparisons were drawn with the ACT genome comparison tool [37]. Identical regions are linked by red connections. Spok genes are in green. Neighboring genes are in light blue and mobile elements in other colors. The red lines depict the different deleted regions. (TIF) [file pgen.1004387.s002.tif]

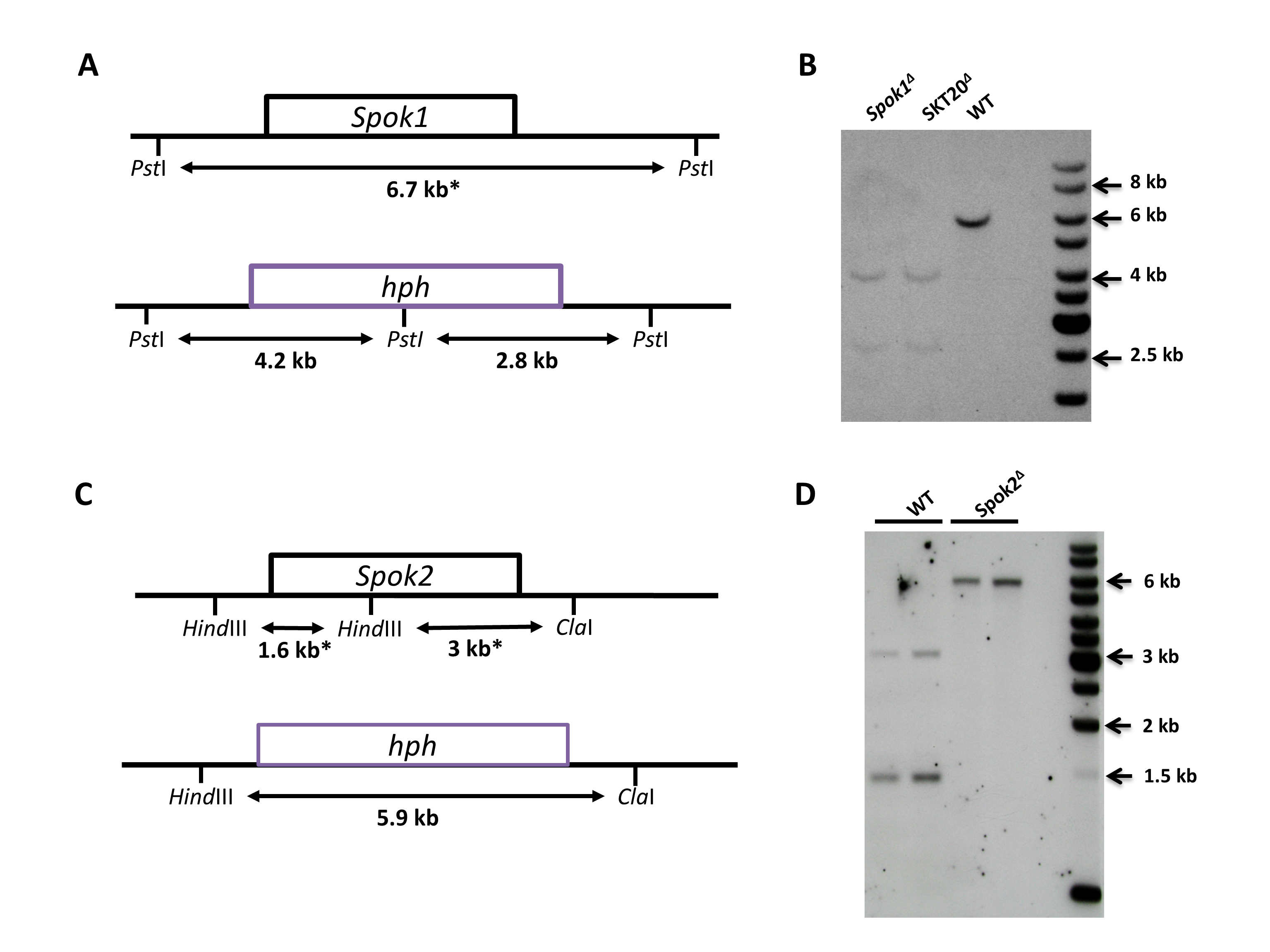

Supplement: Figure S3 — Southern blot analysis of SKT20Δ, Spok1Δ and Spok2Δ strains. Genomic DNA was extracted from the indicated strains and cut with appropriate restriction enzymes. (A) and (C) predicted structures of Spok1 and Spok2 loci before and after marker replacement. (B) and (D) results of Southern blots showing the expected bands. The DNA fragment labeled with * were used as probe. (TIF) [file pgen.1004387.s003.tif]

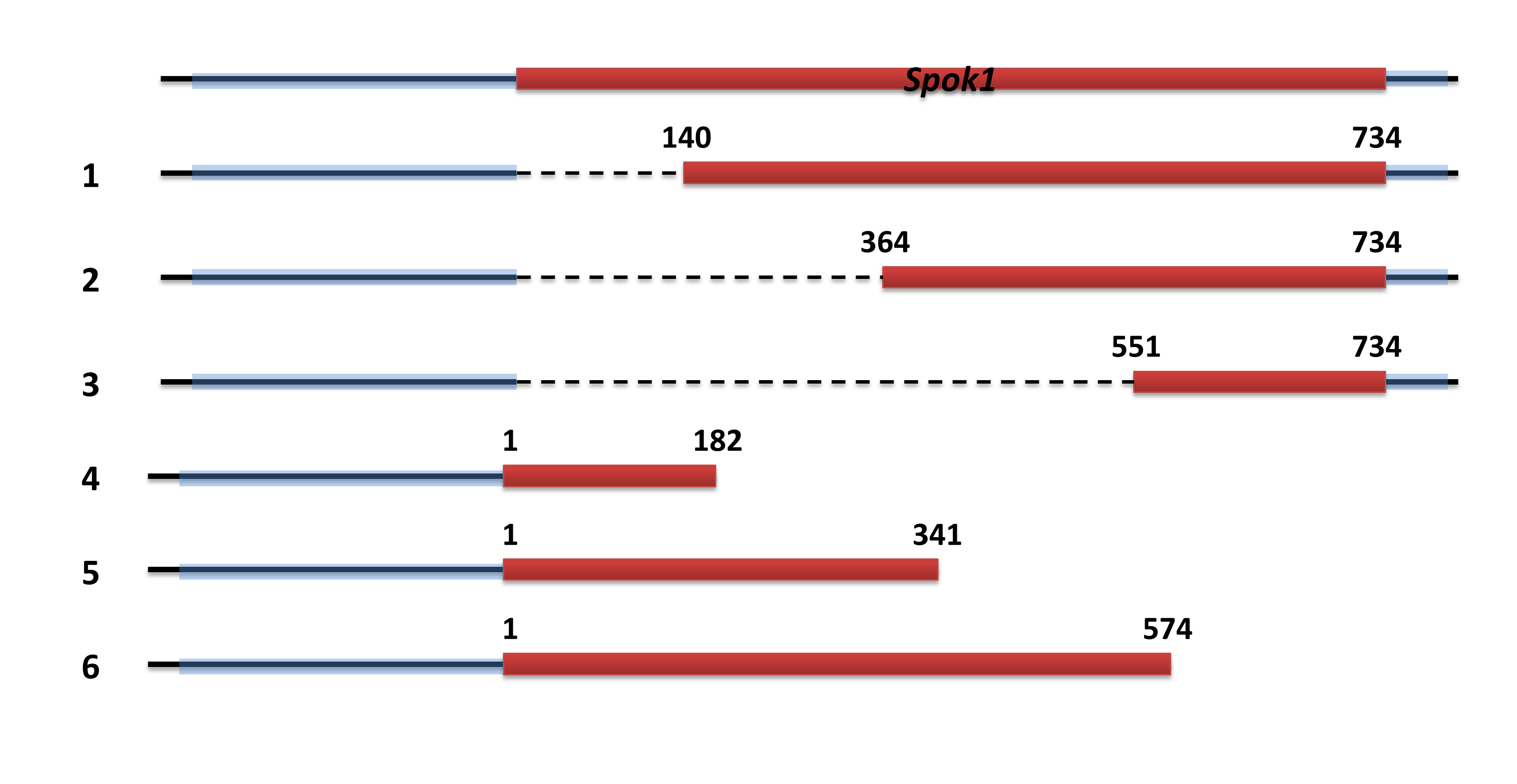

Supplement: Figure S4 — Deletions analysis of Spok1. Schematic representation of the six truncated alleles introduced at the PaPKS1 locus. Codon numbers are indicated. In all constructs, the Spok1 promoter, terminator, start and stop codons were retained. (TIF) [file pgen.1004387.s004.tif]
