# Supplementary material for: Genes That Bias Mendelian Segregation
Source: PLoS Genet. 2014 May 15;10(5):e1004387. doi: 10.1371/journal.pgen.1004387 (PMC4022471; doi:10.1371/journal.pgen.1004387)
Supplement: Table S1 — Strains used in this study. (DOCX) [file pgen.1004387.s005.docx]

**Table S1**

**Strains used in this study**

| Strains | genotypes | *Spok1*^a^ | *Spok2*^a^ | references |
| --- | --- | --- | --- | --- |
| S | wild type reference strain | - | + | ([*1*](#_ENREF_1)) |
| T | wild type reference strain | + | - | ([*2*](#_ENREF_2)) |
| ST1 to ST50 | F1 of S x T | + | ^b^ | ([*3*](#_ENREF_3)) |
| SΔmus51 | *S Δmus51::ble* | - | + | ([*4*](#_ENREF_4)) |
| SΔmus51su8 | *S Δmus51::su8-1* | - | + | ([*4*](#_ENREF_4)) |
| TΔmus51 | *T* *Δmus51::ble* | + | - | This study |
| SKT20 | *S Spok1* | + | + | This study |
| SKT20 ^Δ^ | *S Spok1^Δ^::hph* | - | + | This study |
| Spok1^Δ^ | *T Spok1^Δ^::hph* | - | - | This study |
| Spok2^Δ^ | *S Spok2^Δ^::hph* | - | - | This study |
| SKT20 Spok2 ^Δ^ | *S Spok1 Spok2 ^Δ^::hph* | + | - | This study |
| PaPKS1::Spok1 | *S Δmus51::su8-1 PaPKS1::Spok1,ble* | + | + | This study |
| PaPKS1::Spok2 | *S Δmus51::su8-1 PaPKS1::Spok2,ble* | + | + | This study |

^a^ + gene present, -gene absent

^b^ depending on the strain, *Spok2* may or may not be present see figure S1.

**1. G. Rizet, *Rev. Cytol. Biol. Veg.* 13, 51 (1952).**

**2. E. Padieu, J. Bernet, *C. R. Acad. Sci. Paris* 264, 2300 (1967).**

**3. E. Espagne *et al.*, *Genome biology* 9, R77 (2008).**

**4. K. Lambou *et al.*, *Eukaryotic cell* 7, (Aug 29, 2008).**
