# Supplementary material for: Genes That Bias Mendelian Segregation
Source: PLoS Genet. 2014 May 15;10(5):e1004387. doi: 10.1371/journal.pgen.1004387 (PMC4022471; doi:10.1371/journal.pgen.1004387)
Supplement: Table S2 — Primers used for polymorphic marker analysis, gene deletions and cloning. (DOCX) [file pgen.1004387.s006.docx]

**Table S2**

**Primers used for polymorphic marker analysis, gene deletions and cloning**

| Microsatellite genotyping | | 8-1-1A/B | atcacacgccgaattggt |
| --- | --- | --- | --- |
|  |  |  | gacgccgaagatgctgtc |
|  |  | 8-1-3A/B | agccgaactgtccttaggc |
|  |  |  | gcttggtgcaccttccag |
|  |  | IPH32 | ccgccaattccccataat |
|  |  |  | caccgccactgagggata |
|  |  | 5PH1 | cagggattccgcaggtc |
|  |  |  | gcctcccgacttcttcttg |
|  |  | 5PH3 | atcgcgggtcgactcttt |
|  |  |  | gcacaaaagggctctgga |
|  |  | IPH35 | tgttcgggagtctgaatgg |
|  |  |  | atgccaaccaccaaccac |
|  |  | 5PH6 | tgggtcccttttgtcgag |
|  |  |  | aacccagcaagcagacctt |
|  |  | 5PH5 | tttacggggttgctgcat |
|  |  |  | cccccatttatccgcttac |
|  |  | IPH13 | ggctggagtttgggaaca |
|  |  |  | ctagcatggacaggagcgta |
|  |  | 5PGK | catcccccatcccatgta |
|  |  |  | tttccaccatccggtacg |
|  |  | 5PGM | tccggtgaccctttcttt |
|  |  |  | cgatctgtgttgggcagt |
| 20 kb and 6 kb deletions | 20 kb deletion 1 | Del2A | gagatgaccttggtgatgttcttgtagtcg |
|  |  | Del2B | ctatttaacgaccctgccctgaaccggtcagaaaggtggggtaatctgatgagaac |
|  |  | Del2E | gttctcatcagattaccccacctttctgaccggttcagggcagggtcgttaaatag |
|  |  | Del2F | gcagaacaagtcatttcatttaactcgctgcatcgaactggatctcaacagcggtaag |
|  |  | Del2C | cttaccgctgttgagatccagttcgatgcagcgagttaaatgaaatgacttgttctgc |
|  |  | Del2D | catacacatgcagtcatctggctgaagatac |
|  | 20 kb deletion 2 (containing *Spok1*) | Del3A | ttggcaaaaatattccccttcggcttatac |
|  |  | Del3B | ctatttaacgaccctgccctgaaccgctcggtgactttgtgggttacaagctagag |
|  |  | Del3E | ctctagcttgtaacccacaaagtcaccgagcggttcagggcagggtcgttaaatag |
|  |  | Del3F | cgtctatgacctcttggtaaacatcctcgtcatcgaactggatctcaacagcggtaag |
|  |  | Del3C | cttaccgctgttgagatccagttcgatgacgaggatgtttaccaagaggtcatagacg |
|  |  | Del3D | tcgtataccaaaaaccaccaaatcaaaacc |
|  | 6 kb deletion 1 | Del3A | ttggcaaaaatattccccttcggcttatac |
|  |  | Del3B | ctatttaacgaccctgccctgaaccgctcggtgactttgtgggttacaagctagag |
|  |  | Del4F | gttacacacacaaaagacgcacaaattgatcatcgaactggatctcaacagcggtaag |
|  |  | Del4C | cttaccgctgttgagatccagttcgatgatcaatttgtgcgtcttttgtgtgtgtaac |
|  |  | Del4D | ggtctgatggctcgaggtctgtattatatg |
|  | 6 kb deletion 2 (containing *Spok1*) | Del5A | gacgtagtgttctctaccatggctgtgttc |
|  |  | Del5B | ctatttaacgaccctgccctgaaccgctgtgtgagatggagagcaagtaaaaggtc |
|  |  | Del5E | gaccttttacttgctctccatctcacacagcggttcagggcagggtcgttaaatag |
|  |  | Del5F | gtattaagcacgaggcgtaaaggaatgttgcatcgaactggatctcaacagcggtaag |
|  |  | Del5C | cttaccgctgttgagatccagttcgatgcaacattcctttacgcctcgtgcttaatac |
|  |  | Del5D | ggtatttgattttatttcccgatcggagtc |
|  | 6 kb deletion 3 | Del6A | tataatcctgacctctctcatcaaccatcc |
|  |  | Del6B | ctatttaacgaccctgccctgaaccgtcttggtttcgaaagaaaagccgtattaag |
|  |  | Del6E | cttaatacggcttttctttcgaaaccaagacggttcagggcagggtcgttaaatag |
|  |  | Del3F | cgtctatgacctcttggtaaacatcctcgtcatcgaactggatctcaacagcggtaag |
|  |  | Del3C | cttaccgctgttgagatccagttcgatgacgaggatgtttaccaagaggtcatagacg |
|  |  | Del3D | tcgtataccaaaaaccaccaaatcaaaacc |
| *Spok* genes deletions | Deletion of *Spok2* | DelSPOK2A | gggagacatgttgggttatggaaaaatatg |
|  |  | DelSPOK2B | ctatttaacgaccctgccctgaaccggtcggtctgatggctcgaggtctatatta |
|  |  | DelSPOK2mkE | taatatagacctcgagccatcagaccgaccggttcagggcagggtcgttaaatag |
|  |  | DelSPOK2mkF | gaactcctgtccatctgcttaaaaacgtaccatcgaactggatctcaacagcggtaag |
|  |  | DelSPOK2C | cttaccgctgttgagatccagttcgatggtacgtttttaagcagatggacaggagttc |
|  |  | DelSPOK2D | cgttaatacctttatttttaaggccgggta |
|  | Deletion of *Spok1* | DelSPOK1A | ggtatacaagaaggctgatcttcccagcac |
|  |  | DelSPOK1B | ctatttaacgaccctgccctgaaccggtcggtctgatggctcgaggtctgtattat |
|  |  | DelSPOK1mkE | ataatacagacctcgagccatcagaccgaccggttcagggcagggtcgttaaatag |
|  |  | DelSPOK1mkF | tcttgtccatctgtttaaaaacgtacctcgcatcgaactggatctcaacagcggtaag |
|  |  | DelSPOK1C | cttaccgctgttgagatccagttcgatgcgaggtacgtttttaaacagatggacaaga |
|  |  | DelSPOK1D | caaatttctgctcatcgtaggaacatcact |
| Domains study of *Spok1* | | DomPSI | aaaagtcgacggcgtccataaatagatgctagacctaccg |
|  |  | DomPR1 | ctcgactgcatctacctcaaaacggtctgtgcaatcctgtctttgtcggacatt |
|  |  | DomPR2 | gaaagctggacaggcaaaagctgactagtttgtgcaatcctgtctttgtcggacatt |
|  |  | DomPR3 | atcctttgtagggactcaagagaagctgcttgtgcaatcctgtctttgtcggacatt |
|  |  | Dom1 | aatgtccgacaaagacaggattgcacagaccgttttgaggtagatgcagtcgag |
|  |  | Dom2 | aatgtccgacaaagacaggattgcacaaactagtcagcttttgcctgtccagctttc |
|  |  | Dom3 | aatgtccgacaaagacaggattgcacaagcagcttctcttgagtccctacaaaggat |
|  |  | DomTNI | aaaagcggccgcttagtacagtgctcctgcctgtcgtaacac |
|  |  | Dom4NI | aaaagcggccgctctccgtcatgagctcgcggaggttag |
|  |  | Dom5NI | aaaagcggccgctctccgtcagcaggcacggatgacagt |
|  |  | Dom6NI | aaaagcggccgctctccgtcaagcccatcgctcctctct |
| Integration of *Spok* genes at the centromere of chromosome 2 | *Spok1* | 193SKFSII | aaaaccgcggaggctgcttctggtgtgact |
|  |  | 193SKRSI | aaaagtcgacccttgaccataccagccaac |
|  |  | 510FSI | aaaagtcgacgcgaggaaaacaggacctaa |
|  |  | 510RNI | aaaagcggccgccctgcctgtcgtaacacctt |
|  | *Spok2* | 193SKFSII | aaaaccgcggaggctgcttctggtgtgact |
|  |  | PKSFus | tgacgaaggcttttgttgaaaataccaaaggacagagtcctgtagcaaccctcgtt |
|  |  | Spok2Fus | aacgagggttgctacaggactctgtcctttggtattttcaacaaaagccttcgtca |
|  |  | S510RNI | aaaagcggccgccacggcacggttttcctat |
|  | *Spok^N^* | 193SKFSII | aaaaccgcggaggctgcttctggtgtgact |
|  |  | PKSFusNec | gtgtgaactaccatctcaggaaatacccaggacagagtcctgtagcaaccctcgtt |
|  |  | NecSKFusF | aacgagggttgctacaggactctgtcctgggtatttcctgagatggtagttcacac |
|  |  | NecSKFusR | ctgtgagataagttcttcaagtggtgga |
| PCR detection of *Spok* genes | *Spok* | 510F | accagcgatgggagtttg |
|  |  | 510R | acatccgcgtacgactcc |
|  | *PSS1 / PSST1* | 3950F | agctgctggccttcactg |
|  |  | 3950R | tccccgagcaccgtatc |
|  | *PSS2 / PST2* | 4000F | caacgccgacgagaagag |
|  |  | 4000R | ggcgccttgtactcgatg |
| Construction of Spok2^AA^ | | Spok2MutF | catgggg**c**tg**c**aaatagggccaattatctctgg |
|  |  | Spok2MutR | cctattt**g**ca**g**ccccatgaacaacaccctcg |
